# “I Don't Like to Make a Big Thing out of It”: A Qualitative Interview-Based Study Exploring Factors Affecting Whether Young People Tell or Do Not Tell Their Friends about Their IBD

**DOI:** 10.1155/2020/1059025

**Published:** 2020-06-11

**Authors:** Bernie Carter, Alison Rouncefield-Swales, Lucy Bray, Lucy Blake, Stephen Allen, Chris Probert, Kay Crook, Pamela Qualter

**Affiliations:** ^1^Children, Young People and Families, Faculty of Health, Social Care & Medicine, Edge Hill University, Ormskirk, UK; ^2^Liverpool School of Tropical Medicine, Liverpool, UK; ^3^Institute of Translational Medicine, University of Liverpool, Liverpool, UK; ^4^St. Mark's & Northwick Park, London North West University Healthcare NHS Trust, London, UK; ^5^Manchester Institute of Education, University of Manchester, Manchester, UK

## Abstract

Inflammatory Bowel Disease (IBD) describes a group of conditions that includes Crohn's disease and ulcerative colitis. Unlike some chronic conditions, to a greater or lesser extent, IBD is hidden from or invisible to others which enables concealment of the condition, especially when stigma is associated with the condition. Concealment or nondisclosure allows a means of identity management. Disclosure of a chronic condition is not a single event, and it is dependent on many factors. There is little literature that specifically addresses stigma and/or disclosure in relation to children and young people with IBD. An in-depth qualitative study was undertaken, framed by Interpretive Description and using interviews, friendship maps, and photographs within a participatory framework. Public and patient involvement and engagement (PPIE) was undertaken throughout (inception to dissemination) the study. Young people aged 14-25 years with IBD who had participated in the survey phase of the larger study self-selected to participate in interviews that focused broadly on friendship and feelings of social connectedness. Data were analysed using an iterative, interpretive approach. Preliminary themes were developed and these were explored further, and then tentative theoretical connections about friendship were developed. One superordinate theme focused on disclosure. Thirty-one young people (16 males, 15 females, mean age 18.7 years; 24 Crohn's, 7 colitis) participated in the interviews (of these, five created friendship maps and six utilised photographs). Three discrete, but interlinked, themes were generated, revealing young people's experiences of disclosure: to tell or not to tell; controlling the flow: the who, when, what, and how of telling; and reactions and responses to telling: anticipated and actual. Decisions about telling friends about having IBD are challenging for many young people. Having control over disclosure is not always possible, and the potential consequences can feel risky. However, most young people had positive experiences of disclosure and gained support from friends and romantic partners. Most young people downplayed the seriousness of their IBD, revealing some facets of their condition, aiming to sustain their self-identity. Only one young person had been given professional support to disclose. Provision of support and opportunities to discuss whether, when, who, and how to tell friends and what the risks and benefits may be is something that could be woven into an ongoing and wider person-centred dialogue between young people and health professionals within routine clinic visits.

## 1. Introduction

Inflammatory Bowel Disease (IBD) describes a group of conditions that include Crohn's disease and ulcerative colitis. Up to 25% of IBD starts in childhood or adolescence [1]. IBD is a “chronic, heterogeneous, relapsing and remitting condition primarily as a consequence of inflammation within the bowel lumen” [2]. This group of conditions is characterised by uncertainty, unpredictability, and the intrusiveness of symptoms and flare-ups [3, 4]; common symptoms are diarrhoea, abdominal pain, weight loss, blood in the stools, and fatigue [5]. Both IBD and its associated medical management can severely negatively impact psychosocial functioning and health-related quality of life [6, 7] and increase anxiety and stress [8], loneliness and depression [9, 10], and other psychiatric disorders [11].

Unlike some chronic conditions, to a greater or lesser extent, IBD lacks “surface descriptions” [12]; for the most part, it is hidden from or invisible to others [13], which enables concealment of the condition. People with a hidden condition may decide to conceal it from friends and family and from key stakeholders in their lives such as teachers and employers as a means of identity management [14]. Such concealment helps sustain a sense of self as a “normal” person [15] and avoids negative biographical disruptions [14, 16] such as the stigma and tarnished identity of being known by the bodily disruptions and eruptions of IBD [13]. Young people have to manage those challenges to their identity as they become “emerging adults” [17], a key turning point in their development of self-identity [18].

Stigma is socially constructed, intraindividual, and external [19] and can be defined by characteristics including labelling, separation, stereotyping, status loss, controllability, discrimination, and negative attributions/blame [20, 21]. There are different types of stigma (Table 1). Nondisclosure (deciding not to tell other people) of this aspect of their identity is common among children with IBD [22] because of the ongoing societal taboo about bowel conditions [18]. Disclosure is not a single event [23]: it is a dynamic, selective, complex, context-dependent, and nonlinear progressive journey drawing on multiple strategies [15, 24]; the extent of disclosure can range from partial to full(er) [25]. Who and what a person decides to share about their condition is related to their adjustment to their diagnosis and altered identity [14] and their experiences in different situations such as school, university, or work [26] and their transitions during their life course [13, 24, 27].

Decisions about disclosure are rarely taken lightly because disclosure can be risky [30]. Negative experiences of disclosure can result in psychological distress and poorer quality of life [31]. However, nondisclosure may result in the person experiencing feelings of isolation and psychological distress [26, 32]. The reasons for choosing to disclose are many and varied but include a need for impression management [25], a desire for social support, the wish to promote physical and mental well-being, and the wish to share this aspect of their identity but be in control of that sharing [4, 32]. Other reasons include the desire to educate other people about the condition, support others, and reduce stigma [13]; these reasons are often presented in public campaigns about IBD, such as “it takes guts” (https://www.ittakesguts.org.uk).

Disclosure has been categorised in many different ways, often reflecting the degree of control that the person has over the process. Revelatory or forced disclosure occurs when a degree of openness about their condition is forced on the person regardless of their level of preparedness or their desire to reveal this aspect of their identity [33, 34]. Protective or preventive disclosure is usually selective [34], with the person purposefully disclosing the condition with the aim of gaining control over the social consequences [33]; it may be spontaneous or prompted by a person's enquiry or an unfolding situation [25]. The type and extent of information shared during an episode or episodes of disclosure reflect the purpose of the disclosure but are generally informative about the condition and its consequences [25]; it is often limited and bounded by the need to retain control over privacy and sense of self [13].

Although there is a body of evidence addressing the concepts of stigma and disclosure, most is adult-oriented and/or focused on conditions such as epilepsy and cystic fibrosis or more broadly on chronic conditions. There is little literature [18, 25, 35] that specifically addresses stigma and/or disclosure in relation to children and young people with IBD. The current paper is aimed at contributing to knowledge in this area by presenting qualitative findings on disclosure, generated as part of a large exploratory, sequential mixed method study that explored the impact of IBD on the social relationships and psychological functioning of young people ages 14-25 years, with the condition.

## 2. Study Design

### 2.1. Aim of the Study

The aim of the larger mixed method study —survey (Phase 1) and interviews (Phase 2)—was to explore friendships and feelings of social connectedness among young people with IBD. The analyses presented in this paper are specifically aimed at exploring disclosure and arise from Phase 2 (interviews).

### 2.2. Overview of the Data Collection Methods

The approach used for this in-depth qualitative study was Interpretive Description [36] (a grounded approach that is aimed at answering complex questions about the experience of a condition through articulating patterns and themes generated by interpretation of qualitative data) [37, 38]. Data were collected using interviews, friendship maps, and photographs within a participatory framework.

### 2.3. e-Advisory Group and Wider Engagement with Young People

Our core e-Advisory Group (*n* = 10 young people, 15-26 years old), plus our targeted engagement with young people, parents, and professionals, ensured we were informed and grounded throughout the study. Our engagement with stakeholders provided insight and advice about phrasing of questions within interviews and the potential burden of the surveys and the construction of the findings. This engagement was key to the development of the animation we created to share the young people's experiences of disclosure and disseminate findings.

### 2.4. Sampling, Inclusion Criteria, Recruitment, and Settings

Convenience sampling was used in Phase 1 to recruit young people via invitation letters sent out from hospital clinics, to participate in a survey; recruited young people were able to self-select to participate in the qualitative interview phase of the study. Young people were eligible to participate in this phase of the study if they were aged 14-25 years, had been diagnosed with IBD for at least 3 months, and had completed a Phase 1 survey in either of the two University hospital settings (one children's hospital and one adult hospital) in the North West of England, United Kingdom.

Recruitment for this phase occurred through young people self-identifying at the end of the Phase 1 survey if they were interested in taking part in an interview at their next clinic/hospital visit (providing this fell within the data collection window). The young people who were interested were asked to complete an “expression of interest” form which included their preferred means of being contacted (text, phone, or email) which we used to contact them 2-3 weeks before their scheduled clinic/hospital visit (usually this was 2-3 months following survey completion); if they responded expressing interest, information sheets were sent by post or email. Agreement to meet them at their clinic/hospital visit was made by text/email or phone; consent/assent or a decision to not participate was finalised at that visit and the interview undertaken, as appropriate. Of those who participated in Phase 1 (*n* = 131), 59 provided contact details and were willing to participate in Phase 2, but of these, 28 were unable to participate either due to them not having a scheduled clinic appointment within the timescale of the study, not turning up for their clinic appointment, or researcher unavailability for their specific clinic.

### 2.5. Methods

We utilised three linked data collection methods to provide the young people with a range of different modes of expression because we appreciated that solely asking young people to talk about friendships and IBD might be restricting. We used conversational interviews, friendship maps, and photographs to allow for the generation of both verbal and visual data, with the intention that visual data would trigger conversations and empower young people to have direct control over their interaction with the researcher. The values that underpinned the whole of this study were person-centred and accounted for the sensitivity of the topics we would be discussing; it is our firm belief that young people with IBD are experts of their own experience.

#### 2.5.1. Conversational Face-to-Face Interviews

We adopted a conversational and appreciative approach to the interviews, aiming to ensure that the young people felt comfortable sharing their experiences of friendship and the impact—if any—that IBD had had on those friendships and friendship networks. A sensitively and carefully prepared topic guide with prompts was developed with guidance from young people, the literature, and researcher experience and in collaboration with experts from clinical practice. This guide was aimed at helping the conversation flow. After the first few interviews, we further refined the questions and added some more prompts, reflecting our learning about how the questions worked with the young people in these clinical settings (see supplementary file for revised interview guide (available here)). This also helped to achieve a degree of consistency of questioning and approach across the interviewers. For the interviews that were augmented by the maps and photographs (see later), these materials were woven into the dialogue.

The interviews were conducted face-to-face by four members of the research team who had no responsibility for the young person's care and who were not known to the participants unless they had met them briefly when conducting the earlier survey part of the study. The interviewers were all university-based researchers with expertise in interviewing children and young people; two had backgrounds in children's nursing, one in psychology and one in education. Interviews were conducted in a private room or quiet setting in the clinic/ward during a scheduled and routine visit between October 2018 and April 2019. All interviews were audio-recorded with the young person's permission, and these recordings were then fully transcribed.

#### 2.5.2. Friendship Maps

We asked young people to create a map of their friendships which illustrated who their friends were, how close and strong those friendship ties were, where those friends were situated (e.g., school, clubs), and any losses or overlaps in friendship groups. Typically, short, broad lines reflect close friendships whilst longer, thin lines reflect more tenuous friendships, although explanation by the young person is key to interpretation. The maps could be created prior to or during the interview. These maps could be simple labelled “spider diagrams” or more complex maps including information such as how long they had been friends, where they had met, what made them good friends, and whether or not there had been challenges to these friendships. This approach was based on a participatory mapping technique (PMT) [39, 40], which allows the young person's gaze to be on the map [41] rather than necessarily having to have eye-to-eye contact with the researcher. PMT facilitates a form of reflective sense-making during the creation of the map, allowing the young person time to think through the map they are making and what they wish to share. Being able to revise and refine their maps based on the reflexive dialogue between the participant and the researcher created the opportunities for the process to support emotional reflexivity [42].

#### 2.5.3. Photographs

The use of photographs drew on the photoelicitation technique [43–46] as a means of providing young people with a visual image that they had specifically selected to trigger the conversation and which summed up an aspect of friendship they wished to share.

### 2.6. Ethics Approval

The study was approved by North West-Liverpool East Research Ethics Committee (18/NW/0178) and research ethics committees at Edge Hill University and University of Manchester. Alongside all of the usual care and attention paid to best ethics practice, we developed a “Distress Protocol” in collaboration with the clinical teams, to provide a clear pathway of how we would manage a young person who we believed was distressed either by engagement in the study or who revealed distress/anxiety to us that we felt required support from the clinical team.

### 2.7. Data Analysis

We used an iterative, interpretive approach to data analysis aligned to Interpretive Description [36]. We initially focused on each young person's dataset (interview, friendship map, or photographs) with each interviewer analysing the interviews they had personally undertaken; two researchers (BC and AR-S) worked across all interviews. We initially developed preliminary themes, explored meanings, and developed tentative theoretical connections about disclosure; these were brought together for discussion within the team and challenged and refined. A final consensus set of superordinate themes was generated.

## 3. Findings

### 3.1. Demographics

We interviewed 31 of the 131 young people who participated in the Phase 1 survey. Of these 31, five created friendship maps and six utilised photographs they had taken themselves within their interviews (two gave permission for their photographs to be part of the dataset). The young people who chose to use the additional methods found it helped them talk about issues of importance to them, as it helped them think through the connections and support systems around them. Even when the friendship maps were apparently quite “scribbled and basic” (see supplementary file for example of friendship map (available here)), they acted as starting points and anchors for conversations of importance to them, as did the photographs they shared.

16 participants were males and 15 were females: age at study—mean 18.7 years, range 14-25 years; age at diagnosis—mean 14.4 years, range 8-23 years. Most (*n* = 21) had been diagnosed 5 years or fewer. Twenty-four young people had Crohn's disease (two had stomas) and 7 had colitis (6 with ulcerative colitis and 1 with IBD-unclassified). Seventeen were classified as being in remission, 9 mild, and 5 moderate disease severity and were measured using the appropriate index used in the clinical setting; these were Paediatric Ulcerative Colitis Activity Index (PUCAI), Simple Clinical Colitis Activity Index (SSCAI), Harvey-Bradshaw Index (HB Index), and weighted Paediatric Crohn's Disease Activity Index (wPCDAI) (see Table 2).

The findings presented in this paper focus on disclosure, and this topic was discussed in most detail by the older participants. The findings reveal that the young people with IBD needed a sense of control, albeit illusory and tenuous at times, over the processes linked to disclosure of their condition. Three discrete, but interlinked, themes were generated, revealing young people's experiences of disclosure:

(i) To tell or not to tell

(ii) Controlling the flow: the who, when, what, and how of telling

(iii) Reactions and responses to telling: anticipated and actual

The quotations are presented with key information: interview number (e.g., ID2); gender and age (F12); and condition, e.g., CD or UC or IC.

### 3.2. To Tell or Not to Tell?

At the time of interview, all of the young people had told at least one of their friends something about their condition. For the most part, decisions to tell or not to tell their friends were active, deliberate, and conscious. However, sometimes young people had little or no control because disclosure was forced upon them by particular circumstances, often resulting from treatment and interventions making their condition visible to others. One young man recalled how friends found out when they saw him “drinking this weird pink thing out of a water bottle” (ID7, M14, CD), and disclosure occurred for a young woman as result of her “school [making] a massive deal….[about] the gastric tube” (ID1, F16, CD).

Where young people were able to have control, reasons for not telling related to the desire for “privacy” (ID30, M24, CD) and not wanting “anyone else to know” (ID6, F16, CD). Some decisions not to tell were perhaps less deliberate and more passive; some of the young people simply did not see their condition as something to tell their friends about; “it was just a thing we didn't talk about” (ID1, F16, CD). The “awkwardness of the conversation” (ID12, F23, CD) and the difficulty of explaining the condition made conversations tricky:

*It's hard for to explain colitis, it's the hardest thing in the world… But still now after so many years [to explain] you got ulcerative colitis … it's not just an upset tummy, it's the whole body. And I have lots more complications and I'm on medication. (ID9, F21, UC)*

Embarrassment about the condition was a factor that constrained disclosure. A young woman explained “it's not that I can't tell people, it's that I choose not to because of my own emotions, feelings in general” (ID21, F20, CD), and a young man recalled his embarrassment:

*I think having to say it's colitis, I cannot go out because something might happen... It's not something I want to say. (ID10, M23 UC)*

Other young people reflected that telling friends sometimes involved challenging conversations that they were not always ready for or “in the mood to have” (ID25, M21, CD).

Choosing to tell friends was often a pragmatic decision to be open about their condition, with some young people deciding that “there's no point in trying to hide it” (ID24, M20, CD) or feeling ready to respond openly to an enquiry from friends, “If people ask, I tell them” (ID1, F16, CD), or being prompted to reveal more about their condition at particular times such as during “flare-up” when it was felt important to “mention it to people like my friends” (ID11, F21, UC).

The young people who talked about romantic partners, were mostly 18 years or older and more males than females talked about these relationships. One of the younger girls explained that she was “not really bothered to be honest about boyfriends” (ID17, F15, CD). The young people revealed that the decision to disclose to a romantic partner required particular deliberation; the actual or potential physicality, intimacy, and closeness of their relationships created additional significance. Decision-making about disclosing to actual or potential romantic partners often shifted as the young people got older and/or became more confident about their condition. One young man reflected that his openness grew as he became more relaxed about telling, “I didn't really tell them [at first] but the last two years every girl I've been with I've told them” (ID30, M24, CD).

One young woman explained that her partner “knows everything that is wrong” (ID12, F23, CD). Disclosure was not just limited to what was currently happening but what might happen; one young woman explained she had told her partner about potential IBD-related issues that could arise, such as the need for a stoma, noting “I have had long conversations with him [about possibly having] a colostomy bag” (ID28, F19, CD).

Apart from one young person who had received guidance from her psychologist, none of the young people talked of having received any support from professionals about disclosure.

### 3.3. Controlling the Flow: The Who, When, What, and How of Telling

Apart from wanting control over whether they disclosed their condition, the young people wanted control over who they told, when they told, what they told, and how they told their friends about their condition.

The decision to tell was closely linked with the decision about who to tell, and most of the young people were selective about which of their friends they told; it was sometimes seen as a risky business. They were clear that their IBD was something they “[did not] want everyone to know [about]” (ID6, F16, CD) and that telling was done on a need to know basis; often this selection was based on trust and involved “sussing out who to trust” (ID21, F20, CD) and often resulted in selecting people they considered to be “close friends” (ID30, M24, CD). However, other young people were not selective about who they told, “I told everyone I didn't shy away from it” (ID20, M24, UC) not least because “there's nothing I can do about it [Crohn's]” (ID24, M20, CD). Some were not selective about what they shared:

*I do not mind telling them how often I go to the bathroom either. I have no restrictions. If people want to know, they can. (ID29, F25, CD)*

When the young people were in control of timing disclosure to their friends, typically, this was not immediately after diagnosis; some young people chose to take months or until something triggered telling and others took much longer before telling. One young man “took about two years to even tell the group and I've only told four people” (ID30, M24, CD). Another young person talked of delaying disclosing aspects of their condition even to friends who knew their diagnosis explaining that “if something bad has happened, I will tell a few months afterwards” (ID11, F21, UC). Some did not see that there was an urgency to telling:

*I'm fine with telling friends about it but in a way it's not one of those things that they really care about or in fact I really care about telling them, often I deal with it myself and with my family. (ID3, M15, CD)*

The content of what young people disclosed varied depending on factors such as their confidence in explaining the condition, the level of detail they wanted to reveal, and the purpose behind telling their friends.

Many of the young people reported that they found it “kind of like hard to explain” their condition in a way that their friends would understand, especially when they were unaware of the level of knowledge of or misconceptions about the condition their friends might have. Typically, they avoided going “into complete detail” (ID30, M24, CD) about their condition because they “don't like to make a big thing out of it” (ID6, F16, CD). They often aimed to elicit an undramatic response from their friends:

*Yeah, I just told them the story of what happened really and they were just like, it's good that you cope with it. (ID2, F14, CD)*

Many of the descriptions downplayed the seriousness of the condition and/or the severity of symptoms, and some talked of how they “just laughed off” (ID 19, M22, CD) or “make a joke about it” (ID11, F21, UC) or masked even quite troubling symptoms as they “didn't want to make a fuss….. be defined by it” (ID10, M23, UC). The language chosen to describe their condition was often carefully selected “I tend to just use generic terms, "My stomach's not feeling very well or things like that" (ID24, M20, CD).

The reason why the young people were disclosing impacted the content of what they told their friends. Some were simply explaining that they had the condition, some disclosed as a means of gaining support, and others had a more educative focus, wanting to their friends to better understand what their condition encompassed:

*They [friends] associate you with wanting to go to the toilet, and I'm like, ‘No, it's not always about that'. There's a lot of different aspects, like, it goes from your mouth, like, to your bum, it goes all the way down, and so it's just trying to get that across. (ID18, F25, CD)*

However, for some young people, the reason for disclosure was to warn potential friends of what they might be getting into, with one recently diagnosed young woman suggesting that disclosure allowed their friends to “decide whether or not it's worth them investing time [in me]” (ID21, F20, CD).

Having decided who and what to tell, young people had to decide how to tell their friends. The “how” of telling involved considering whether to tell face-to-face or remotely as well as issues such as where to tell. Few young people talked of having guidance or information from professionals. Only one young person talked about gaining professional support about how to disclose, “I started seeing my psychologist and then we came up with ideas on how to tell them” (ID17, F15, CD). Most young people told their friends face-to-face, although some told remotely “When they [doctors] said ‘You've got Crohn's' - I texted them [friend]” and the young person who had spoken to her psychologist “made a snap chat group so I could tell them all at once rather than doing it separately and still having to say the same thing” (ID17, F15, CD). Others used a mix of face-to-face and the Internet, relying on the Internet to fill in gaps of understanding, “It's when people say what is that and I'm like, ok, google it, yeah” (ID21, F20, CD).

### 3.4. Reactions and Responses to Telling: Anticipated and Actual

Decisions about disclosure were not without consequences, and although for most of the young people disclosure was not a negative experience, some young people's fears about being rejected or seen as different were fulfilled. One young person explained that “some people think that because it's a disease, Crohn's - they say ‘Oh my God can I catch it off you?'” (ID1, F16, CD). Some young people recalled their initial overriding anxiety about talking about their condition, even if they were unsure why they were anxious, explaining “so I didn't want to speak to anyone I didn't know... I was just concerned about like… I don't know really” (ID8, M14, IC).

Some young people were concerned that even if responses were not overtly negative, their friends might be thinking about them negatively, “they don't necessarily have to react badly, but if I think that they're thinking something then…” (ID12, F23, CD). Others were worried about being pitied, “can't be bothered with [pity]” (ID28, F19, CD) or their condition becoming the focus of attention:

*I've got a colostomy bag – it'll turn into a discussion all about that and, the sort of, the fear is always … I do not want the sympathy... I feel getting sympathy from people and people sort of viewing me as ‘different' or ‘not well' when in myself I am well,…..could, in my view, make them maybe see less of me as a person. (ID25, M21,CD)*

Mostly, young people were surprised by the support they gained from friends, even when they had been expecting rejection, “I was like, ‘I've got this' and they were like, ‘Oh' and supportive of me and stuff” (ID, 26, M16, UC). Another young man talked of the support he gained from a friend who also has a chronic condition.

*Like he's got diabetes and stuff so he has to be careful with drinking and stuff, that sort of thing. And he said he knows someone who's got colitis and stuff. (ID10, M23 UC)*

The ability to “open up” to supportive friends who knew and understood was described as being “quite heartening” with young people knowing “I can tell them stuff in private and they wouldn't tell people or judge me for it” (ID13, F16, UC).

Several young people talked of the importance of their friends knowing about their condition as it helped in “forging friendships” and allowed them to “feel a lot closer to someone if I know that they know exactly what I'm going through” (ID20, M24, UC). Another young person found that their condition created unexpected connections:

*People sort of got along with me even better because I probably was feeling a bit low and probably come out with more outrageous [funny] stuff, so the Crohn's business…like having to go for a colonoscopy, having to go for an endoscopy …, silly stories to tell people. (ID22, M21, CD)*

Some people had been selective about who they had told, and that had led to some awkward situations when “some people know things that others don't…. it gets a bit awkward then because you've got to like, explain” (ID25, M21, CD).

Other young people were much more sanguine about the potential or actual loss of friends as a result of disclosing their condition, explaining they had been “lucky,” as mostly their friends had been “relatively accepting apart from a few who I've just cut out of my life because I thought they're not worth being friends with” (ID28, F19, CD).

In the context of romantic relationships, the stakes were often seen to be higher and young people balanced the anxiety of disclosing with the consequences of unexpectedly cancelling dates due to illness:

*I do not tell them [girlfriends] at first but if it's like the third date and I want to keep seeing them ….. I just want to kind of just tell them and for them to understand because there can be times where I cancel a date at the last minute. (ID30, M24, CD)*

Other young people acknowledged that sharing information was more problematic for them than their partners with one young man explaining how he “kind of like tiptoe around and things like that especially if, you know, I'm having toilet troubles” (ID24, M20, CD) whereas his partner is “OK” about it.

## 4. Discussion

Within the current study, issues related to disclosure featured in most of the stories shared by the young people. Three key processes were presented in the findings: whether or not to tell their friends; who, when, what, and how to tell; and the responses to telling. Each of those aspects was closely interlinked. For example, the degree of confidence or concern about how friends might respond to being told impacted whether young people decided to disclose and to whom they disclosed. This discussion draws on findings about disclosure from both IBD-specific and other chronic conditions and spans literature addressing children and adults. Little of this literature focuses exclusively on disclosure to friends.

Disclosure to friends should perhaps be better described as a series of disclosures prompted by changes in the young person's life that generate new opportunities to make friends such as changing school and moving to college or university or into the workplace. As reported elsewhere, disclosure was clearly a dynamic, complex, and context-dependent process; it was not a one-off event [23, 24, 27, 30–32]. Young people's desire for control of disclosure was evident in relation to each of the key processes. However, disclosure, much like their condition, was not something over which they had full control. Across this diverse sample (age at diagnosis, years of living with condition, age at interview, and gender), little difference was noted between the young men and the young women in terms of who they told (e.g., same or different sex friends), when, or how they told.

IBD is uncertain, unpredictable, and intrusive [3, 4]. Our findings support other work on IBD [25] and conditions such as epilepsy [23] that disclosure is a process laced with uncertainty and unpredictability [18, 25] particularly in relation to the potential intrusive consequences. In our study, few young people talked of having guidance, support, or information from professionals or accessing resources to help them prepare for or deal with what was initially perceived as being the risky business of disclosure. Some form of personal disclosure plan might be of value; such an approach has been shown to be useful for disclosure to employers by young people with mental illness [47]. “Practice telling” or rehearsal has been shown to be helpful for young people with epilepsy [15]. Individuals had different disclosure experiences; some more positive than others [47]. All the young people in the current study had been living with a diagnosis of IBD for at least 3 months, and all of them had disclosed to at least one friend. Young people decided to disclose when the time felt right for them, and as noted elsewhere, it was part of their “processing journey” [15] and the particular situation or setting [30] and life stage [24]. Fundamentally, this reflected their sense of self or self-identity [17] and their level of comfort in disclosing, as also seen in disclosure by young people with epilepsy [15]. As noted elsewhere, for some young people, disclosure was a “prominent issue” [48]; for others, either the prominence had faded or it was less of an issue for them. Either way, it would seem useful if guidance was offered in relation to how to tell.

We found that there were different reasons for nondisclosure, and these were similar to findings from other studies on disclosure: the young people in the current study were also fearful of being seen as “less of a person” [15] or being perceived as different or tarnished [4, 13], pitied [26], rejected [32, 34], or teased [22]. Others were concerned about being morally judged by others [18] or wanted to keep knowledge of their condition private [13]. Unlike some conditions, the invisibility of IBD allowed the young people to try and sustain their preillness identity, positioning themselves as healthy [49], thus avoiding the potential labelling, discrimination, and loss of status and control that can occur with a stigmatising illness [21, 22].

Many of the young people shared, even if in passing, the potential for their illness to stigmatise. The influence of cultural stigma was evident in that the young people were aware that bowel conditions or talking about bowels is still something of a societal taboo [18]. This sets up the context in which some young people talked of an expectation that friends would or might stigmatise them (anticipated stigma) [19]. Others talked of concerns that their friends might feel stigmatised because of their connection with them (associative stigma) [19]. Anticipated stigma became enacted stigma for some young people when established friendships or friendship networks were damaged or lost by disclosure, as seen elsewhere [14]. Unexpected responses included friends worrying if the condition was catching; contagion is also associated with stigma (associative stigma). Although some young people were concerned to a lesser or greater degree about their condition tarnishing their identity [13], none of them talked in a way that suggested they accepted that discrimination against them would be deserved (internalised stigma). When stigma was felt, anticipated, or enacted, the response was to perceive that any labelling or damage to friendships reflected a weakness or shallowness in the person or people doing the labelling or cutting friendship ties.

For the most part, the young people in the current study took the decision to disclose seriously, and that has also been reported by other young people with IBD [35]. Most exercised a cautious and deliberate approach to the process, and as in other studies, they weighed the potential benefits and costs [24] and were selective [42] about which friend or friends they talked to about their condition. They acknowledged that not everyone needed to know [15] and were purposive about what they shared. Findings from other studies support our own conclusions that the young people initially tended to select close friends whom they thought they could trust [25, 34]; however, sometimes they were disappointed by their friends' reactions to their disclosure.

Reasons for telling their friends varied within the current sample. Often, telling friends reflected a desire to be honest or open [4] with their friends, to let them know what was happening or explain their absences or changes in behaviour [13], and those reasons were similar regardless of whether they were disclosing to everyday friends or potential/actual romantic friends or partners. This form of protective [30, 50] or preventive disclosure [33] is evident in other chronic conditions such as epilepsy [15, 23, 34], where the desire to disclose is informed by the need to establish control over perceived negative social consequences such as rejection or being stigmatised. In addition, that control can sustain their self-identity and be a form of impression management [14, 25]. A minority of young people in the current study were more open about their condition with their friends, apparently unconcerned about the potential consequences or unbothered about dealing with them if they arose.

Some young people experienced forced and unplanned [34, 51] disclosure, a situation in which they had little or no control as something or someone else had revealed their condition, either intentionally or unintentionally [33, 34]. Those young people had little or no time to prepare themselves for telling and talked of feeling exposed by this. In these situations, their ability to conceal their IBD, because of its lack of “surface descriptions” [12, 25] failed, and their IBD was made public. In the current study, the intentional broadcasting [34, 51] of the young person's condition by a school at morning assembly was almost certainly done with the best of motives, but this was not the young person's perception.

As seen in other studies, disclosure conversations about their condition with friends ranged from being easy to awkward or challenging [25], partly because of the uncertainty [25] about, and unpredictability of, their friends' initial and ongoing responses. The decisions about what and how to share reflected the young person's experience in disclosure and what they had learned in the process both about themselves and other people's reactions and where they were on their life journey. As they gained experience in telling, they had a clearer idea of what they wanted to say and the extent of disclosure. As seen elsewhere, perceived risks were somewhat higher when disclosing to an actual/potential romantic friend or if the relationship was or likely to be intimate [52], but in these cases, it was pragmatically often impossible not to disclose [26]; the salience of the condition is raised in such situations [53]. Typically, the young people tended towards partial rather than full disclosure [25], revealing generic, less embarrassing aspects that were likely to elicit the least dramatic responses and which were less awkward to share. As seen elsewhere, the seriousness of their condition was often downplayed [13]; this was achieved by using simple language and explanations. Even when telling, boundaries were in play; other work with children with IBD notes their tendency to restrict disclosure to limited information about the condition [25]. Limiting explanations reflected a desire to both protect themselves and shelter their friends from too much detail—a sense of the more you tell, the more you risk. Typically, the young people told their friends face-to-face, the approach most usually reported for disclosure; however, as seen in other studies, some used social media [17, 26].

Although some authors suggest that a key purpose of disclosure is to elicit support [13, 32], this was less overt in this study. However, the support of friends was often a very positive outcome of having the courage to tell them about their condition, as friends were able to accommodate the young person's needs and “be there” for them [14].

Despite the challenges associated with disclosure, most of the young people in the current study managed this without professional advice or support [52]; little literature addresses this key issue. Most were also apparently unaware of the resources that might have been helpful. However, most IBD resources are not focused on disclosure and young people, although CCUK's recently developed Talking Toolkit (https://www.ittakesguts.org.uk/talk/talking-toolkit) is likely to be of value. Indeed, findings from an experimental study on stigma and IBD found a positive correlation between an increase in knowledge of IBD and reduced stigma [29]. The young people in our study and the members of our e-advisory group recognised the need for a resource developed from their experiences and ideas. The “Telling My Friends” resource (a two-minute animation and information sheets aimed at supporting young people in telling their friends and at helping their friends support them) is the end result of collaborative working (https://ehu.ac.uk/CrohnsorColitis). Our hope is that our contribution to increasing awareness of IBD may help to reduce the stigma and taboo associated with the condition.

### 4.1. Limitations of the Study

The sample for this qualitative phase of the study was recruited using convenience sampling from Phase 1 and from two hospitals within the same city and was not ethnically diverse, with most being White. Thus, our findings may not be representative of the wider population. In addition, the views of young people with colitis were less present than those with a diagnosis of Crohn's disease. Those with severe disease activity chose not to participate in this phase.

## 5. Conclusion

The findings outlined in this paper are part of a larger study addressing friendships and social connection: disclosure was a key aspect that the young people talked about. It seems clear that decisions about telling friends about having IBD are challenging for many young people. Provision of support and opportunities to discuss whether, when, who, and how to tell friends and what the risks and benefits may be is something that could be woven into an ongoing and wider person-centred dialogue between young people and health professionals within routine clinic visits. The young person's IBD nurse may have a particularly important role to play here in terms of their ongoing engagement with them. This dialogue could focus not only on those things of direct relevance to the clinician's clinical management of the young person's condition but also on those things linked to their condition that matter to the young person, such as disclosure.

## Figures and Tables

**Table 1:** Types of stigma (developed from [19, 28, 29]).

Type of stigma	Description
Anticipated	Expectation by the person with condition that people will stigmatise them
Perceived or felt	Degree to which person perceives stigma directed at them from others
Internalised	How much the person believes that discrimination against them is deserved
Enacted	Social discrimination by people towards those with condition
Associative or affiliate	Stigma experienced because of connection with a stigmatised person
Kinship	Being or feeling stigmatised by members of family
Cultural or external	Degree to which society/culture person living in devalues person's condition

**Table 2:** Key demographics of participants.

ID	Gender	Age yrs (age diagnosed)	Diagnosis	Surgery (stoma)	Disease activity^1,2,3,4^
2	Female	14 (11)	Crohn's	No	Mild^4^
4	Male	14 (13)	Crohn's	No	Remission^4^
7	Male	14 (12)	Crohn's	No	Mild^4^
3	Male	15 (9)	Crohn's	Yes (stoma)	Remission^4^
5	Male	15 (9)	Crohn's	Yes	Mild^4^
16	Male	15 (11)	Crohn's	No	Remission^4^
17	Female	15 (12)	Crohn's	No	Moderate^4^
27	Female	15 (11)	Crohn's	No	Remission^4^
1	Female	16 (11)	Crohn's	No	Mild^4^
6	Female	16 (13)	Crohn's	No	Remission^4^
14	Female	16 (10)	Crohn's	Yes	Remission^4^
15	Male	16 (15)	Crohn's	No	Remission^4^
28	Female	19 (8)	Crohn's	Yes	Remission^3^
21	Female	20 (19)	Crohn's	No	Remission^3^
24	Male	20 (12)	Crohn's	No	Mild^3^
31	Female	20 (12)	Crohn's	Yes	Moderate^3^
23	Male	21 (14)	Crohn's	No	Remission^3^
25	Male	21 (20)	Crohn's	Yes (stoma)	Remission^3^
19	Male	22 (21)	Crohn's	Yes	Remission^3^
12	Female	23 (15)	Crohn's	Yes	Mild^3^
22	Male	24 (16)	Crohn's	No	Moderate^3^
30	Male	24 (19)	Crohn's	No	Remission^3^
18	Female	25 (23)	Crohn's	Yes	Mild^3^
29	Female	25 (23)	Crohn's	Yes	Mild^3^
13	Female	16 (15)	Ulcerative colitis	No	Remission^1^
26	Male	16 (12)	Ulcerative colitis	No	Mild^1^
9	Female	21 (8)	Ulcerative colitis	No	Remission^2^
11	Female	21 (16)	Ulcerative colitis	No	Moderate^2^
10	Male	23 (20)	Ulcerative colitis	No	Moderate^2^
20	Male	24 (22)	Ulcerative colitis	No	Remission^2^
8	Male	14 (12)	Unclassified colitis	No	Remission^1^

Key: ^1^Paediatric Ulcerative Colitis Activity Index (PUCAI); ^2^Simple Clinical Colitis Activity Index (SSCAI); ^3^Harvey-Bradshaw Index (HB Index); ^4^weighted Paediatric Crohn's Disease Activity Index (wPCDAI).

## Data Availability

Consideration of data availability will be made on a case by case basis.

## Supplementary Materials

Supplementary Materials Supplementary File 1: questions from Friendship Interview Guide (revised version). Supplementary File 2: example of friendship map.

## References

[1] Benchimol E. I., Fortinsky K. J., Gozdyra P., van den Heuvel M., van Limbergen J., Griffiths A. M. (2011). Epidemiology of pediatric inflammatory bowel disease: a systematic review of international trends. *Inflammatory Bowel Diseases*.

[2] Ashton J. J., Harden A., Beattie R. M. (2018). Paediatric inflammatory bowel disease: improving early diagnosis. *Archives of Disease in Childhood*.

[3] Roberts C. M., Gamwell K. L., Baudino M. N. (2019). Youth and parent illness appraisals and adjustment in pediatric inflammatory bowel disease. *Journal of Developmental and Physical Disabilities*.

[4] Nicholas D. B., Otley A., Smith C., Avolio J., Munk M., Griffiths A. M. (2007). Challenges and strategies of children and adolescents with inflammatory bowel disease: a qualitative examination. *Health and Quality of Life Outcomes*.

[5] Ashton J. J., Cullen M., Afzal N. A., Coelho T., Batra A., Beattie R. M. (2018). Is the incidence of paediatric inflammatory bowel disease still increasing?. *Archives of Disease in Childhood*.

[6] Stapersma L., van den Brink G., van der Ende J. (2019). Illness perceptions and depression are associated with health-related quality of life in youth with inflammatory bowel disease. *International Journal of Behavioral Medicine*.

[7] Kim S., Park S., Kang Y., Kwon N., Koh H. (2018). Medical staff tend to underestimate the quality of life in children and adolescents with inflammatory bowel disease. *Acta Paediatrica*.

[8] Jelenova D., Prasko J., Ociskova M. (2016). Quality of life and parental styles assessed by adolescents suffering from inflammatory bowel diseases and their parents. *Neuropsychiatric Disease and Treatment*.

[9] Loftus E. V., Guérin A., Yu A. P. (2011). Increased risks of developing anxiety and depression in young patients with Crohn's disease. *The American Journal of Gastroenterology*.

[10] van den Brink G., Stapersma L., el Marroun H. (2016). Effectiveness of disease-specific cognitive–behavioural therapy on depression, anxiety, quality of life and the clinical course of disease in adolescents with inflammatory bowel disease: study protocol of a multicentre randomised controlled trial (HAPPY-IBD). *BMJ Open Gastroenterology*.

[11] Butwicka A., Olén O., Larsson H. (2019). No association of childhood-onset inflammatory bowel disease with risk of psychiatric disorders and suicide attempt. *Gut*.

[12] Moss P., Dyck I. (2003). *Women, Body, Illness: Space and Identity in the Everyday Lives of Women with Chronic Illness*.

[13] Defenbaugh N. L. (2013). Revealing and concealing ill identity: a performance narrative of IBD disclosure. *Health Communication*.

[14] Kirk S., Hinton D. (2019). "I'm not what I used to be": a qualitative study exploring how young people experience being diagnosed with a chronic illness. *Child: Care, Health and Development*.

[15] Pembroke S., Higgins A., Pender N., Elliott N. (2017). Becoming comfortable with "my" epilepsy: strategies that patients use in the journey from diagnosis to acceptance and disclosure. *Epilepsy & Behavior*.

[16] Bray L., Kirk S., Callery P. (2014). Developing biographies: the experiences of children, young people and their parents of living with a long-term condition. *Sociology of Health & Illness*.

[17] Ravert R. D., Crowell T. L. (2008). ‘I have cystic fibrosis’: an analysis of web-based disclosures of a chronic illness. *Journal of Clinical Nursing*.

[18] Saunders B. (2014). Stigma, deviance and morality in young adults' accounts of inflammatory bowel disease. *Sociology of Health & Illness*.

[19] Quinn D. M., Chaudoir S. R. (2015). Living with a concealable stigmatized identity: the impact of anticipated stigma, centrality, salience, and cultural stigma on psychological distress and health. *Stigma and Health*.

[20] Link B. G., Phelan J. C. (2001). Conceptualizing stigma. *Annual Review of Sociology*.

[21] Bresnahan M., Zhuang J. (2011). Exploration and validation of the dimensions of stigma. *Journal of Health Psychology*.

[22] Hommel K. A. (2013). Psychosocial and behavioral issues in children and adolescents with IBD: clinical implications. *Gastroenterology & hepatology*.

[23] Kilinç S., Campbell C. (2009). "It shouldn't be something that's evil, it should be talked about": a phenomenological approach to epilepsy and stigma. *Seizure*.

[24] Werner S., Halpern A., Kurz S., Rosenne H. (2019). Disclosure in cystic fibrosis: a qualitative study. *Journal of Social Issues*.

[25] Barned C., Stinzi A., Mack D., O’Doherty K. C. (2016). To tell or not to tell: a qualitative interview study on disclosure decisions among children with inflammatory bowel disease. *Social Science & Medicine*.

[26] Kaushansky D., Cox J., Dodson C., McNeeley M., Kumar S., Iverson E. (2016). Living a secret: disclosure among adolescents and young adults with chronic illnesses. *Chronic Illness*.

[27] Lindsay S., Cagliostro E., Carafa G. (2018). A systematic review of disability disclosure & accommodations for youth in post-secondary education & employment. *Archives of Physical Medicine and Rehabilitation*.

[28] Chaudoir S. R., Quinn D. M. (2010). Revealing concealable stigmatized identities: the impact of disclosure motivations and positive first disclosure experiences on fear of disclosure and well-being. *The Journal of Social Issues*.

[29] Rohde J. A., Wang Y., Cutino C. M. (2017). Impact of disease disclosure on stigma: an experimental investigation of college students’ reactions to inflammatory bowel disease. *Journal of Health Communication*.

[30] Micallef-Konewko E. (2013). Talking about an invisible illness: the experience of young people suffering from inflammatory bowel disease (IBD). *School of Psychology*.

[31] Oliver K. N. (2011). *Perceived stigma and self-disclosure in adolescents and adults living with cystic fibrosis: measuring the impact on psychological and physical health*.

[32] Hughes S. L., Romo L. K. (2020). An exploration of how individuals with an ostomy communicatively manage uncertainty. *Health Communication*.

[33] Tröster H. (1997). Disclose or conceal? Strategies of information management in persons with epilepsy. *Epilepsia*.

[34] Benson A., O'Toole S., Lambert V., Gallagher P., Shahwan A., Austin J. K. (2015). To tell or not to tell: a systematic review of the disclosure practices of children living with epilepsy and their parents. *Epilepsy & Behavior*.

[35] Varni J. W., Shulman R. J., Self M. M. (2017). Patient health communication mediating effects between gastrointestinal symptoms and gastrointestinal worry in pediatric inflammatory bowel disease. *Inflammatory Bowel Diseases*.

[36] Thorne S. E. (2016). *Interpretive Description : Qualitative Research for Applied Practice*.

[37] Teodoro I. P. P., Rebouças V. . C. F., Thorne S. E., Souza N. K. M. ., Brito L. S. A. ., Alencar A. M. P. G. (2018). Interpretive description: a viable methodological approach for nursing research. *Escola Anna Nery*.

[38] Thorne S., Con A., McGuinness L., McPherson G., Harris S. R. (2016). Health care communication issues in multiple sclerosis: an interpretive description. *Qualitative Health Research*.

[39] Emmel N. (2008). Participatory mapping: an innovative sociological method real life. *Methods*.

[40] Involve (2010). *Using participatory mapping to explore participation in three communities*.

[41] Campbell S., Clark A., Keady J. (2019). Participatory social network map making with family carers of people living with dementia. *Methodological Innovations*.

[42] Burkitt I. (2012). Emotional reflexivity: feeling, emotion and imagination in reflexive dialogues. *Sociology*.

[43] Harper D. (2002). Talking about pictures: a case for photo elicitation. *Visual Studies*.

[44] Carter B., Ford K. (2013). Researching children's health experiences: the place for participatory, child-centered, arts-based approaches. *Research in Nursing & Health*.

[45] Ford K., Bray L., Water T., Dickinson A., Arnott J., Carter B. (2017). Auto-driven photo elicitation interviews in research with children: ethical and practical considerations. *Comprehensive Child and Adolescent Nursing*.

[46] Wells F., Ritchie D., McPherson A. C. (2013). ‘It is life threatening but I don't mind’. A qualitative study using photo elicitation interviews to explore adolescents' experiences of renal replacement therapies. *Child: Care, Health and Development*.

[47] McGahey E., Waghorn G., Lloyd C., Morrissey S., Williams P. L. (2016). Formal plan for self-disclosure enhances supported employment outcomes among young people with severe mental illness. *Early Intervention in Psychiatry*.

[48] Greene K., Magsamen-Conrad K., Venetis M. K., Checton M. G., Bagdasarov Z., Banerjee S. C. (2012). Assessing health diagnosis disclosure decisions in relationships: testing the disclosure decision-making model. *Health Communication*.

[49] Spencer G., Lewis S., Reid M. (2017). Living with a chronic health condition: students’ health narratives and negotiations of (ill) health at university. *Health Education Journal*.

[50] Charmaz K. (2016). Stories and silences: disclosures and self in chronic illness. *Qualitative Inquiry*.

[51] Elafros M. A., Mulenga J., Mbewe E. (2013). Peer support groups as an intervention to decrease epilepsy-associated stigma. *Epilepsy and Behavior*.

[52] Heller M. K., Gambino S., Church P., Lindsay S., Kaufman M., McPherson A. C. (2016). Sexuality and relationships in young people with spina bifida and their partners. *Journal of Adolescent Health*.

[53] Kelly M. (1992). Self, identity and radical surgery. *Sociology of Health and Illness*.

