# Supplementary material for: “I Don't Like to Make a Big Thing out of It”: A Qualitative Interview-Based Study Exploring Factors Affecting Whether Young People Tell or Do Not Tell Their Friends about Their IBD
Source: Int J Chronic Dis. 2020 Jun 11;2020:1059025. doi: 10.1155/2020/1059025 (PMC7305549; doi:10.1155/2020/1059025)
Supplement: Supplementary Materials — Supplementary File 1: questions from Friendship Interview Guide (revised version). Supplementary File 2: example of friendship map. [file 1059025.f1.docx]

**Supplementary File 1: Questions from Friendship Interview Guide (revised version).**

Please tell me a bit about yourself

- Home/family?
- School, college, university, work etc?
- What do you enjoy doing?

If you were writing down a list of friends, who would be on that list

- Tell me something about those friends
- What sort of things do you usually do with these friends?
- What do you enjoy most about being with these friends?

If you were taking a picture or pictures or imagining a picture(s) that summed up you and your friends what would that picture(s) be of?

Do you have a friend to whom you feel closest? Or are all of your friendships equally close?

- For those who have a best friend: How long have you been friends with one another?
- What do you value most about this friendship?

Do you have different groups of friends?

- How long have you known them?
- How did you meet them?

What is the thing you value most in a friendship?

- Is it spending time together, laughing at the same kind of things, being able to tell them things? Or all those things?

Do you find any aspect of your friendships difficult or challenging?

- Like telling your friends about how you feel? Or finding time to spend with them?
- Do you work hard at your friendships?

Do you think having IBD has had an impact on your friendships?

- Do you think it has an impact on the way that you make friends? Or keeping friendships going?
- Did anything in your friendships change when you found out you had IBD?
  - If yes, what kind of things? In what kind of ways did the friendship change?
- Do things change with your friends when/if your IBD gets worse?
  - If yes, in what kind of ways?

Would you like to change anything about your current friendships?

- If yes, what kind of things?

Do you see anything in the future that makes you worried about your friendships?

- If yes, what kind of things? (e.g. leaving home)?

Anything else about your friendships that you want to tell us about? Anything that I didn’t ask?

**Supplementary File 2: Example of Friendship Map**

This young woman struggled to disclose and had felt rejected by friends who either did not understand or who had made a few off-putting comments. On the map, LD is her ‘best friend’ and RM is her long term on/off boyfriend, both of whom understand and are supportive. OH and AB are friends who she felt have drifted away due to issues in not understanding
